# CyaY and TusA regulate ISC- and SUF-mediated l-cysteine desulfurase activity†

**DOI:** 10.1039/d4cb00225c

**Published:** 2024-09-27

**Authors:** Paolo Olivieri, Jason C. Crack, Angelika Lehmann, Nick E. Le Brun, Silke Leimkühler

**Affiliations:** a Institute of Biochemistry and Biology, Department of Molecular Enzymology, University of Potsdam D-14476 Potsdam Germany sleim@uni-potsdam.de +49-331-977-5128 +49-331-977-5603; b Centre for Molecular and Structural Biochemistry, School of Chemistry, Pharmacy and Pharmacology, University of East Anglia Norwich Research Park Norwich NR4 7TJ UK N.Le-brun@uea.ac.uk

## Abstract

CyaY, the frataxin homolog of *Escherichia coli*, plays an important role in ISC iron–sulfur cluster assembly through interactions with the cysteine desulfurase IscS, which regulate the supply of sulfur. IscS is not exclusive for ISC Fe–S cluster assembly, as it functions as a hub for the supply of sulfur to a number of other sulfur-requiring pathways, such as for the biosynthesis of Moco and thiolated tRNAs. How the balance of sulfur supply to the various competing pathways is achieved is not fully understood, but a network of protein–protein interactions plays a key role. For example, IscU and TusA compete for binding to IscS and thus for sulfur supply to ISC and Moco/tRNA biosynthesis. Here, we show that TusA can displace CyaY from IscS and can form hetero-complexes involving IscS, CyaY and TusA. Displacement of CyaY from IscS raised the question of whether it can interact with the SUF pathway. The SUF cysteine desulfurase SufS functions as a complex with SufE. Native mass spectrometry studies showed that the SufS dimer can bind up to four SufE molecules, two at high affinity, and two at low affinity, sites. Titration of SufSE (or SufS alone) with CyaY demonstrated binding, probably at the lower affinity site in competition with SufE. Binding of CyaY dramatically reduced the activity of SufSE *in vitro*, and over-expression of CyaY also significantly affected total cellular desulfurase activity and Fe–S cluster assembly, with the greatest effect observed in mutant strains in which SufS was the principal desulfurase. These data point to a physiological role for CyaY in regulating the desulfurase activity of IscS and SufS and, hence, both the *E.coli* iron–sulfur assembly systems. They also demonstrate that TusA can displace the regulatory CyaY protein from IscS–CyaY complexes, facilitating sulfur delivery from IscS to other essential cellular processes, and increasing the likelihood of SufSE–CyaY interactions.

## Abstract 2

CyaY, the frataxin homolog of *Escherichia coli*, is known to regulate ISC iron–sulfur cluster assembly through binding to IscS. It also interacts with the SUF system, through binding to, and attenuating activity of, SufS.

## Graphical abstract

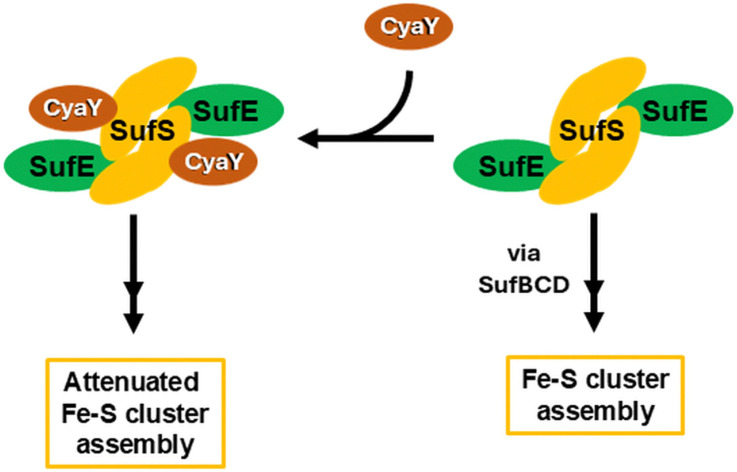

## Introduction

Iron–sulfur (Fe–S) clusters are evolutionary ancient prosthetic groups.^1^ The assembly of Fe–S clusters is carried out by highly conserved biosynthetic machinery, which is encoded by the *suf* and *isc* operons in most bacteria.^2^ The *E. coli* ISC system is encoded by the *iscRSUA-hscBA-fdx-iscX* operon,^3^ with IscR being the transcriptional regulator of the system, that in its [2Fe–2S]-cluster-bound form represses the expression of the operon.^4^ IscS and IscU are essential components of the ISC machinery:^5^ IscS is an l-cysteine desulfurase that provides sulfur (as sulfane, S^0^) in the form of a protein-bound persulfide and transfers it further to IscU,^6^ the scaffold protein on which the Fe–S clusters are assembled. Assisted by the chaperones HscAB^7,8^ the newly formed cluster is released and further transferred onto IscA,^9^ an A-type carrier protein that itself transfers the cluster to target proteins.^10^ Fdx interacts with IscS and provides reducing equivalents for persulfide reduction/cluster formation.^11^

IscS also interacts with several other proteins, including CyaY, which is not encoded by the *isc* operon.^12,13^ Frataxin (FXN), the human homologue of CyaY, plays an important role in assembly of Fe–S clusters and regulation of iron homeostasis in mitochondria.^14^ In *E. coli*, CyaY was shown to regulate the activity of IscS by slowing down the rate of Fe–S cluster formation. In contrast, FXN was shown to activate Fe–S cluster assembly in mitochondria in eukaryotes.^15^ This contrasting effect, however, was shown to depend on the nature of the l-cysteine desulfurase.^15^

IscX, which is encoded by the *isc* operon, interacts with Fe^2+^, IscU and IscS, but its exact role is less well understood than those of other proteins of the ISC machinery.^16^ Recently, it was shown that CyaY and IscX compete for the same binding site on IscS, with IscX acting as a modulator of CyaY, switching off its inhibitory influence in response to the iron concentration.^17^ At low iron concentrations, IscX binds to IscS, while at higher iron concentrations CyaY principally binds to IscS.^17^

The *E. coli* SUF system, encoded by the *sufABCDESE* operon, is activated under conditions unfavorable to Fe–S cluster stability and biogenesis, such as iron starvation or oxidative stress.^18^ The SUF system, which is under the regulation of IscR and Fur,^19^ includes SufSE, an l-cysteine desulfurase complex,^20^ SufA, an A-type carrier protein,^9^ and SufBC_2_D, a scaffold complex.^21^

In addition to IscU, Fdx, CyaY, and IscX, which are involved in Fe–S cluster assembly, several additional proteins were identified as interaction partners of IscS, including TusA and ThiI. TusA is required for Moco biosynthesis and the mnm^5^s^2^U34 thiomodification in tRNAs, while ThiI is involved in both thiamine biosynthesis and s^4^U8 tRNA modifications.^22^ Overall, there is a complex protein–protein interaction network focused around IscS, as the master enzyme in the initial mobilization of sulfur from l-cysteine and its transfer, in the form of a persulfide, onto specific sulfur acceptor proteins.^23^ So far, the interaction sites of IscU, ThiI, TusA, IscX, Fdx and CyaY have been mapped on IscS.^22^ Previous studies predicted that the IscS partner proteins bind to IscS only one at a time.^24^ The exception seems to be the Fe–S assembly process that involve the formation of a ternary complex, consisting of IscS–IscU and either CyaY, Fdx or IscX.^17,25^ Early studies suggested that IscU could displace TusA from a complex with IscU.^22^ More recently, the competition between TusA and IscU for IscS has been more quantitatively explored, confirming the preference of IscS for IscU,^26^ and implying that Fe–S cluster assembly may be prioritized under conditions of sulfur limitation.

While apparent prioritisation may be the ultimate outcome, it is likely an oversimplification of how sulfur transfer is regulated.^23,27^ Deletion of *tusA* caused a pleiotropic effect on several cellular pathways in *E. coli*, including enhanced susceptibility of viral infection inhibition by programmed ribosomal frameshifting, in addition to tRNA thiolation and Moco biosynthesis.^28^ These pleiotropic effects were suggested to be caused by changes in the Fe–S cluster concentration in the cell, implying a link between Fe–S cluster availability and tRNA thiolation and Moco biosynthesis.^29^ Furthermore, elevated levels of TusA in *E. coli* decreased the level of Fe–S clusters. Consequently, when Fe–S clusters are limited, Fe–S containing proteins such as MoaA are inactive, resulting in a decrease in activity of molybdoenzymes. Surprisingly, overexpression of IscU also reduced the level of active molybdoenzymes in *E. coli*,^29^ possibly because of an elevated complex formation of IscU with IscS, thereby limiting IscS availability for interaction with other proteins such as TusA.^29^

The recent observation of hetero-complexes involving the IscS dimer and single IscU and TusA molecules, and the resulting effects on IscS l-cysteine desulfurase activity led to the proposal of a model in which the delivery of sulfur to different sulfur-requiring pathways is controlled by sulfur acceptor protein levels, IscS binding affinities, and acceptor protein-modulated IscS desulfurase activity.^26^

In this study, we have extended our investigation of how protein–protein interactions contribute to the regulation of Fe–S cluster assembly. Through native mass spectrometry and *in vitro* and *in vivo* activity assays, we show that TusA has a higher affinity for IscS than does CyaY and can displace it. With IscS unavailable, CyaY can also bind to SufS or the SufSE complex, leading to dramatic inhibition of SufS desulfurase activity *in vitro* and *in vivo*. This points to a regulatory effect of both TusA and CyaY on Fe–S cluster biosynthesis, *via* interactions with both IscS and SufS.

## Results

### IscS preferentially binds TusA over CyaY

Structures of IscU–IscS and TusA–IscS complexes have revealed that IscS contains two distinct but adjacent, binding sites for IscU and accessory proteins (*e.g.* Fdx, IscX, TusA, and CyaY)^17,22,24,27^ TusA and IscU binding sites on IscS partially overlap, but the TusA binding site is otherwise comparable to the binding site utilized by other accessory proteins (*e.g.* Fdx, IscX and CyaY), suggesting CyaY and TusA compete for binding to IscS. Mass spectrometry under non-denaturing (native) conditions can provide direct information on protein complexes present in solution.^30–33^ Using this approach, we recently showed that IscU displaces TusA from complexation with IscS, consistent with structural data, and that IscU and TusA form mixed hetero complexes on the IscS dimer, with one side binding IscS and the other TusA.^26^ It was of further interest to investigate the competitive binding of CyaY and TusA to IscS.

The *m*/*z* spectrum of dimeric IscS displayed well-resolved charge states, as previously observed.^17,30,34^ The deconvoluted spectrum of IscS revealed a major peak at 90 662 Da (predicted mass: 90 642 Da + Na = 90 663 Da), consistent with the presence of dimeric IscS and associated PLP cofactors, as previously described.^30^

**Fig. 1:**
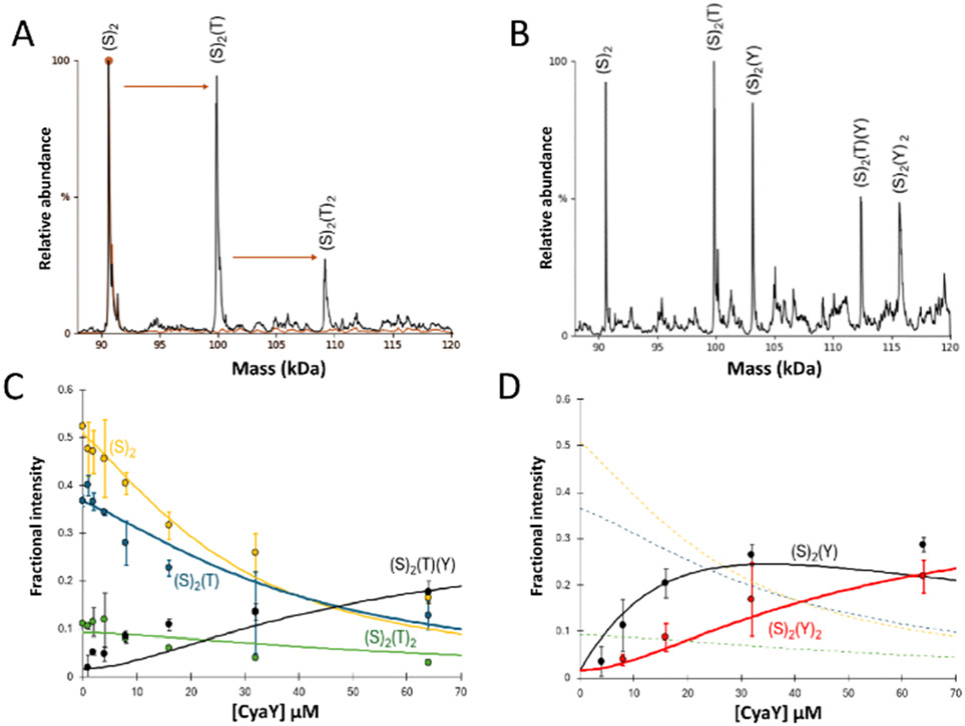
ESI-MS investigation of complex formation in mixtures containing IscS, TusA and CyaY. (A) Deconvoluted mass spectrum of IscS over the mass range 88–125 kDa, showing the presence of the IscS (red spectrum). Addition of TusA at a 0.5 : 1 ratio gave rise to TusA–IscS complexes in which the IscS dimer is bound by one or two TusA protein molecules (black spectrum). (B) Deconvoluted mass spectra of an identical TusA–IscS sample, following the addition CyaY at a 4 : 1, revealing a CyaY–TusA–IscS hetero-complex. (C) and (D) Plots of relative intensity of relevant complexes, as indicated, as a function of CyaY concentration. Solid lines show fits of the data to a simple competitive binding model for 1–2 TusA per IscS dimer. Dashed lines in (D) are shown for comparison and correspond to TusA–IscS species shown in (C). IscS and subsequent complexes were in 250 mM ammonium acetate, pH 8. Note that abundances in (A) and (B) are reported relative to the most abundant species, which is arbitrarily set to 100%.

Initially, IscS (8 μM IscS, 4 μM dimeric IscS) was pre-treated with 0.5 equivalents of TusA (4 μM) to generate TusA–IscS complexes (Fig. 1(A)).

The predominant species in the native MS spectrum was (TusA)(IscS)_2_ together with (IscS)_2_ and (TusA)_2_(IscS)_2_ complexes. An equivalent sample was then treated with 4 equivalents (16 μM) of CyaY to see which ternary complexes might form. Homo-complexes of CyaY–IscS ((CyaY)(IscS)_2_ and (CyaY)_2_(IscS)_2_), TusA–IscS ((TusA)(IscS)_2_) together with the hetero-complex (CyaY)(TusA)(IscS)_2_ were observed (Fig. 1(B)). No evidence for the binding of >2 accessory proteins (CyaY and/or TusA) per IscS dimer was observed, consistent with the idea that CyaY and TusA utilise similar binding sites on IscS, and cannot utilise the additional, lower affinity site that was detected for IscX.^17^

To further investigate the effect of CyaY, the TusA–IscS complexes were titrated with increasing amounts of CyaY. This resulted in the gradual formation of (CyaY)(TusA)(IscS)_2_, (CyaY)(IscS)_2_ and (CyaY)_2_(IscS)_2_, and concomitant decline of (TusA)(IscS)_2_, (TusA)_2_(IscS)_2_ and (IscS)_2_ complexes. The (CyaY)(IscS)_2_ and (CyaY)(TusA)(IscS)_2_ complexes were detectable at [CyaY]/[IscS] ≈ 0.5, while (CyaY)_2_(IscS)_2_ was detectable by [CyaY]/[IscS] ≈ 1. The (CyaY)(IscS)_2_ complex maximized at [CyaY]/[IscS] ≈ 4, but neither (CyaY)(TusA)(IscS)_2_ nor (CyaY)_2_(IscS)_2_ complexes maximized during the titration. The data were analysed according to a simple competition binding model (Fig. 1(C) and (D)). The resulting fit was satisfactory, revealing that complex formation is governed by the higher affinity of TusA for IscS, with dissociation constant (*K*_d_) = ∼6 μM, compared to that of CyaY for IscS (*K*_d_ = ∼26 μM).

These values are consistent with previous reports of CyaY binding to IscS with a *K*_d_ of ∼23 μM^27^ and a *K*_d_ for the TusA–IscS complex of ∼8 μM.^29^ Reciprocal experiments, in which pre-formed CyaY–IscS complexes were titrated with TusA, confirmed the binding preference of IscS for TusA over CyaY (Fig. S1, ESI†).

### The SufS dimer can bind up to four SufE molecules

**Fig. 2:**
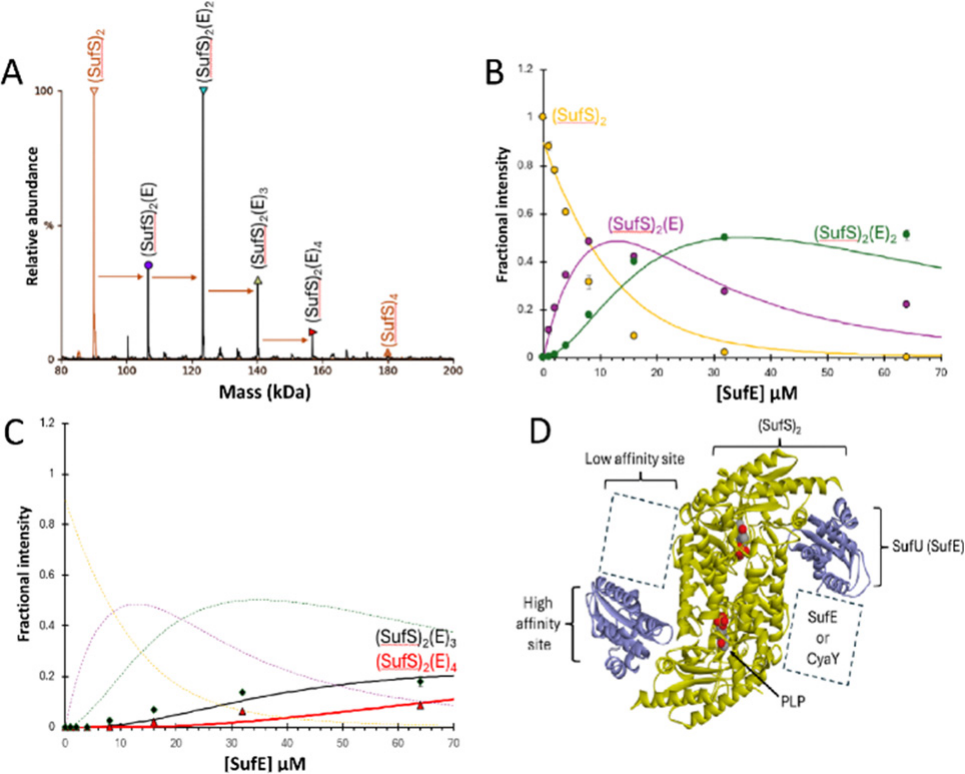
ESI-MS investigation of complex formation between SufS and SufE. (A) Deconvoluted spectrum of SufS before (red spectrum) and after (black spectrum) the addition of SufE at a 4 : 1 ratio, indicating the formation of multiple SufE–SufS complexes, as indicated. (B) and (C) Plots of relative intensity of various complexes, as indicated, as a function of SufE concentration. Solid lines show fits of the data to a simple binding model, with pairs of dissociation constants for 1–2 and 3–4 TusA per IscS dimer. Dashes lines in (C) are shown for comparison and correspond to species shown in (B). (D) Cartoon of SufE–SufS complex, indicating possible location of high and low affinity binding sites, based on the structure of *B. subtilis* SufU–SufS (PDB: 5XT5).^5,22,38–40^ SufS and subsequent complexes were in 250 mM ammonium acetate, pH 8. Note that abundances in (A) are reported relative to the most abundant species, which is arbitrarily set to 100%.

The deconvoluted mass spectrum of SufS revealed a major peak at 89 956 Da (predicted mass: 89 892 Da + (S^0^)_2_ = 89 956 Da), consistent with the presence of dimeric SufS and associated PLP cofactors. Like IscS, SufS readily forms complexes with additional proteins.^2,35–37^ The addition of SufE to dimeric SufS, to give an 8 : 1 molar ratio, resulted in significant changes in the *m*/*z* spectrum (Fig. S2, ESI†). A new pattern of charge states, superimposed over those of residual SufS, were observed, consistent with the presence of multiple SufE–SufS complexes with up to 4 SufE molecules, at intervals of ∼16.7 kDa, interacting with SufS (Fig. 2(A)), reminiscent of IscX–IscS interactions.^17^

When increasing amounts of SufE were added to SufS, the gradual formation of SufS complexes containing 1 to 4 SufE molecules was observed. The complex with a single SufE, (SufE)(SufS)_2_, formed readily at low levels of SufE ([SufE]/[SufS] ≈ 0.1) and maximized at [SufE]/[SufS] ≈ 1.

The (SufE)_2_(SufS)_2_ complex was detectable at [SufE]/[SufS] ≈ 0.5 and reached maximum abundance by [SufE]/[SufS] ≈ 4 (Fig. 2(B)). The (SufE)_3_(SufS)_2_ complex reached maximum abundance at [SufE]/[SufS] ≈ 8, while the (SufE)_4_(SufS)_2_ complex did not reach maximum abundance during the titration (Fig. 2(C)). The data were analysed according to a sequential binding model. The resulting fits of the data revealed that binding of the first two SufE molecules, to form (SufE)(SufS)_2_ and (SufE)_2_(SufS)_2_, occurred with a similar affinity, *K*_d_ = ∼8 μM. Binding of the third and fourth SufE molecules (to give (SufE)_3_(SufS)_2_ and (SufE)_4_(SufS)_2_) occurred with a significantly lower affinity, *K*_d_ = ∼71 μM (Fig. 2(B) and (C)). Thus, SufS contains two distinct binding sites for SufE, with one primary (high affinity) and one secondary (low affinity) site per SufS monomer. In contrast, TusA does not bind to SufS (see Fig. S3, ESI†), consistent with previous observations.^41^

### SufSE complexes can bind CyaY

Blaenburg *et al.* previously showed that *B. subtilis* frataxin (CyaY) is capable of binding to the *B. subtilis* SufU–SufS complex *via* an adjacent binding site.^39^ To determine if the *E. coli* proteins might also interact, SufSE complexes were treated with a 4-fold excess of CyaY.

**Fig. 3:**
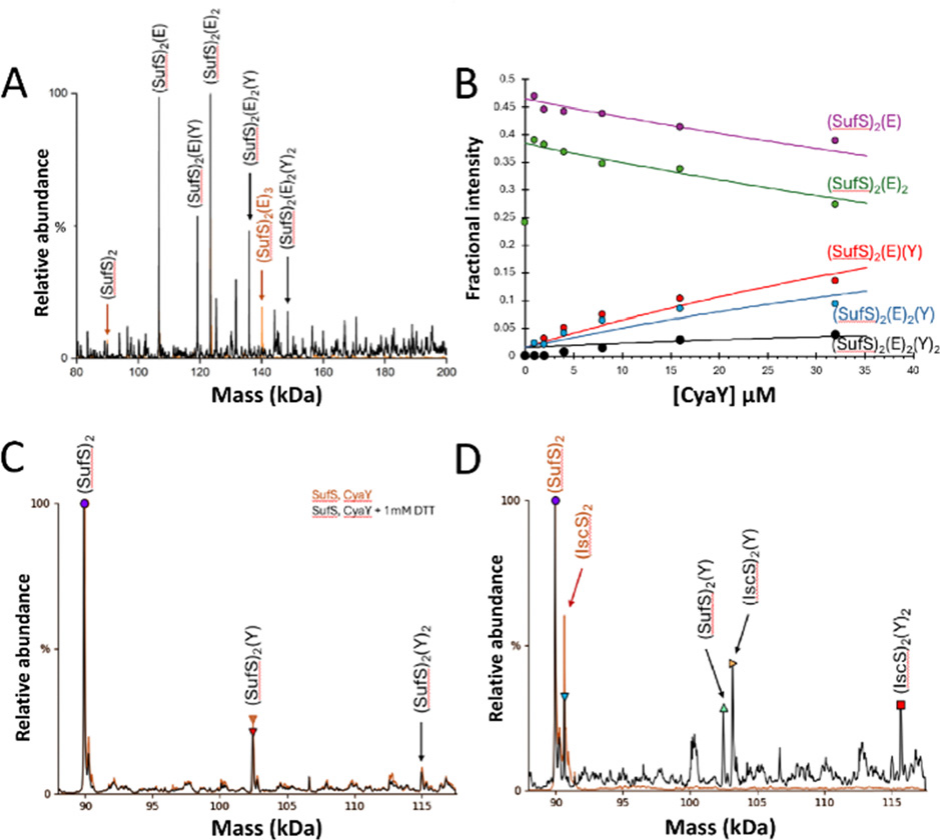
ESI-MS investigation of complex formation between SufE, SufS and CyaY. (A) Deconvoluted spectrum of SufE–SufS complexes before (red spectrum) and after (black spectrum) the addition of CyaY at a 4 : 1 ratio gives multiple CyaY–SufE–SufS complexes, as indicated. (B) Plots of relative intensity of relevant complexes, as indicated, as a function of CyaY concentration. Solid lines show fits of the data to a simple binding model, with pair of dissociation constants. (C) CyaY–SufS complexes in the presence (black line) and absence (red line) of 1 mM DTT, indicating that the interaction is not dependent on SufE nor non-physiological disulfide bond formation. (D) CyaY–SufS complexes still form in the presence of equal molar amounts of IscS and SufS, despite the preference of CyaY for IscS.

The predominant species in the native MS spectrum were (SufE)(SufS)_2_, and (SufE)_2_(SufS)_2_, with modest contributions from (CyaY)(SufE)(SufS)_2_ and (CyaY)(SufE)_2_(SufS)_2_, and trace amounts of a potential (CyaY)_2_(SufE)_2_(SufS)_2_ complex (Fig. 3(A)).

To further investigate CyaY binding, SufSE complexes were titrated with increasing amounts of CyaY, Fig. 3(B). The data suggest that CyaY can interact with SufSE complexes but does so with relatively low affinity. The data clearly represent only the initial part of a titration curve(s), with saturation of binding not achieved over the concentration range investigated. At higher CyaY concentrations (>35 μM), the presence of intense charge states from CyaY multimers (CyaY_*n*= 2–3_) were observed to cause ion suppression, interfering with the deconvolution process (Fig. S4, ESI†) and preventing the exploration of higher CyaY concentrations. It is well known that frataxin (CyaY) may, under certain conditions, form higher order globular complexes with itself or other proteins,.^42–49^ Fitting of the data gave an estimation of the *K*_d_ as ∼70 μM (but note this is an approximation because only the first part of the titration was measured), consistent with the stability being lower than for the corresponding interaction of CyaY with IscS (*K*_d_ ≈ *ca.* 23 μM).^31^

To determine if the interaction of CyaY with SufS is dependent upon the presence of SufE, comparable experiments were conducted in the absence of SufE. Again, (CyaY)(SufS)_2_ and (CyaY)_2_(SufS)_2_ complexes were observed, indicating SufE is not a prerequisite for CyaY binding. These complexes also formed in the presence or absence of 1 mM DTT, ruling out artefactual disulfide-bonded complexes (Fig. 3(C)).

Finally, an 8-fold excess of CyaY was added to equimolar amounts of IscS and SufS (8 μM each, 4 μM dimeric species). The interaction of CyaY with IscS generated the expected (CyaY)(IscS)_2_ and (CyaY)_2_(IscS)_2_ complexes, together with a small proportion of the (CyaY)(SufS)_2_ complex, increasing the likelihood that the CyaY–SufSE complexes reported above may be physiologically relevant (Fig. 3(D)).

### *In vitro* l-cysteine desulfurase activity

**Fig. 4:**
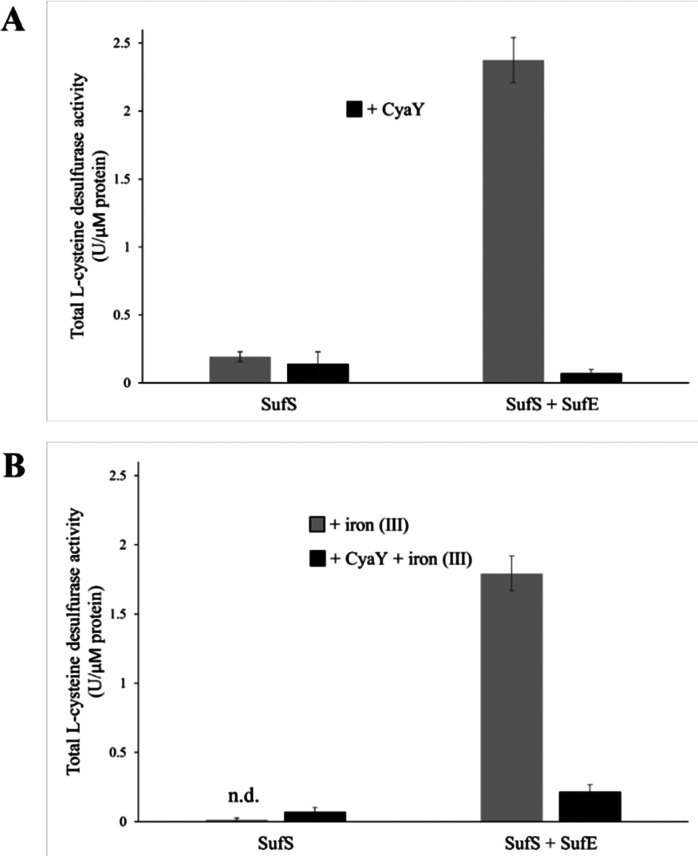
Influence of CyaY on the l-cysteine desulfurase activity of SufS and SufSE *in vitro*. The effect of CyaY on the l-cysteine desulfurase activity of SufS and SufSE was quantified after the release of sulfide as methylene blue. The assay was carried out under different conditions: (A) in absence (grey bars) or presence of CyaY (black bars) or (B) in absence (grey bars) or presence of CyaY (black bars) with the addition of 50 μM FeCl_3_. Here, 30 μM of SufS or 30 μM SufS plus 90 μM SufE were incubated, with or without 90 μM of CyaY and/or 50 μM of FeCl_3_, for 10 min at 30 °C in the presence of 1 mM DTT and 1 mM l-cysteine. One unit is defined as the amount of enzyme producing 1 μmol of sulfide/min. Error bars are derived from at least 6 independent measurements, n.d.: no activity detectable.

To test the effect of CyaY binding to SufS, the l-cysteine desulfurase activities of SufS and the SufSE complex were analyzed, in the presence or absence of additional iron (by the addition of FeCl_3_), to test the effect of iron, since the SUF operon is only expressed under iron-limiting conditions^18^ (see below). In the absence of SufE, the l-cysteine desulfurase activity of SufS was very low (Fig. 4(A)). The addition of CyaY to SufS did not significantly affect desulfurase activity. The SufSE complex exhibited significant desulfurase activity, which was virtually abolished upon addition of CyaY (Fig. 4(A)).

Equivalent assays in the presence of Fe^3+^ yielded broadly similar results. The activity of SufS/CyaY was even lower than in the absence of additional Fe^3+^. The presence of iron also somewhat inhibited desulfurase activity of SufSE, while addition of CyaY reduced activity by ∼90%. Thus, the inhibitory effect of CyaY was somewhat less pronounced in the presence of additional Fe^3+^.

### *In vivo* l-cysteine desulfurase activity

To further investigate the effect of CyaY on the activity of SufS, *in vivo*l-cysteine desulfurase activities of wild-type *E. coli* (BW25113 parental strain) and Δ*iscS*, Δ*sufS*, Δ*tusA*/Δ*iscS* and Δ*tusA*/Δ*sufS* mutant strains, with and without over-expression of the *cyaY* gene, were investigated.

**Fig. 5:**
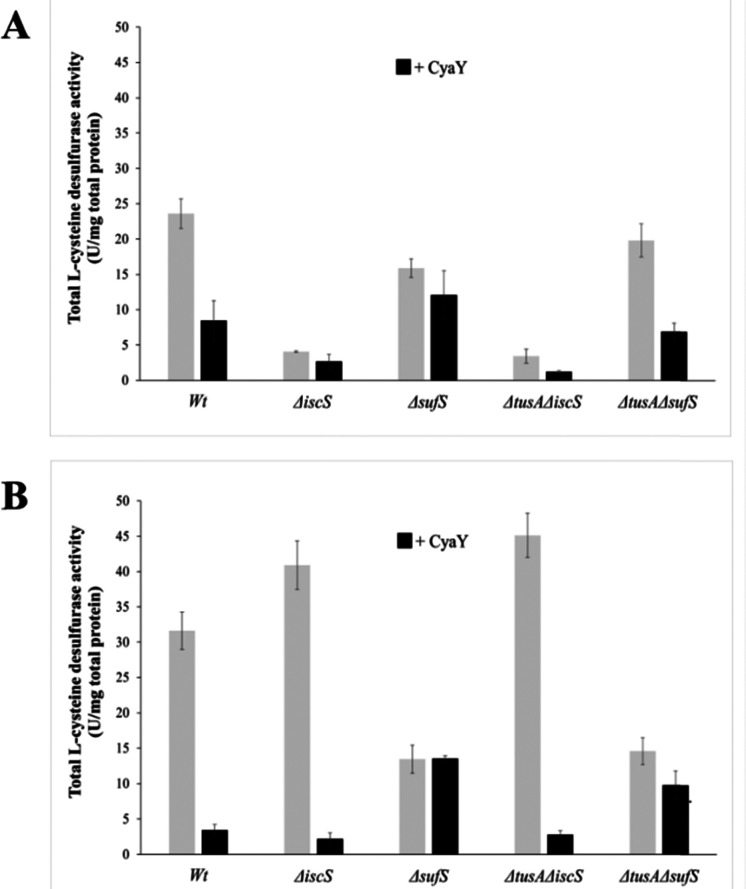
Influence of CyaY on the total H_2_S production from cell extracts of different *E. coli* strains. The total H_2_S production of *E. coli* strains BW25113 (wild-type), Δ*iscS* Δ*sufS* and Δ*tusA*Δ*iscS*, and Δ*tusA*Δ*sufS* was measured in crude extracts following the methylene blue assay. The different *E. coli* mutant strains were cultivated 7 h under aerobic conditions (A) before (grey bars) or after the expression of CyaY (black bars) or low iron (B) before (grey bars) or after the expression of CyaY (black bars) in the presence of 100 μM of 2,2′-dipyridyl to reduce the available iron concentration. The expression of CyaY was induced by addition of 20 μM IPTG to the growth medium. Error bars are derived from at least 6 independent measurements.

As can be seen in Fig. 5, the largest effect of CyaY was observed in the presence of dipyridyl, conditions under which the *suf* operon would be expressed in *E. coli*. After additional expression of CyaY, the l-cysteine desulfurase activity dropped by ∼90% in the BW25113 strain, and by >90% in the Δ*iscS* strain, in which the principal desulfurase activity is from SufS. Similarly, a ∼90% decrease in activity was observed upon over-expression of CyaY in the Δ*tusA*Δ*iscS* strain, which also measures mainly the activity of SufS.

In the Δ*sufS* and Δ*tusA*Δ*sufS* strains, in which the l-cysteine desulfurase activity was mainly due to IscS, the presence of CyaY had a much less severe effect on activity. However, the effect of CyaY on Δ*sufS* mutant strains, and particularly the Δ*tusA*Δ*sufS* mutant, was more pronounced under iron-replete conditions. This is consistent with the proposal that an iron-bound form of CyaY binds more tightly to IscS, resulting in inhibition of l-cysteine desulfurase activity of IscS,^17^ and that TusA can displace CyaY from IscS (Fig. 1).

**Fig. 6:**
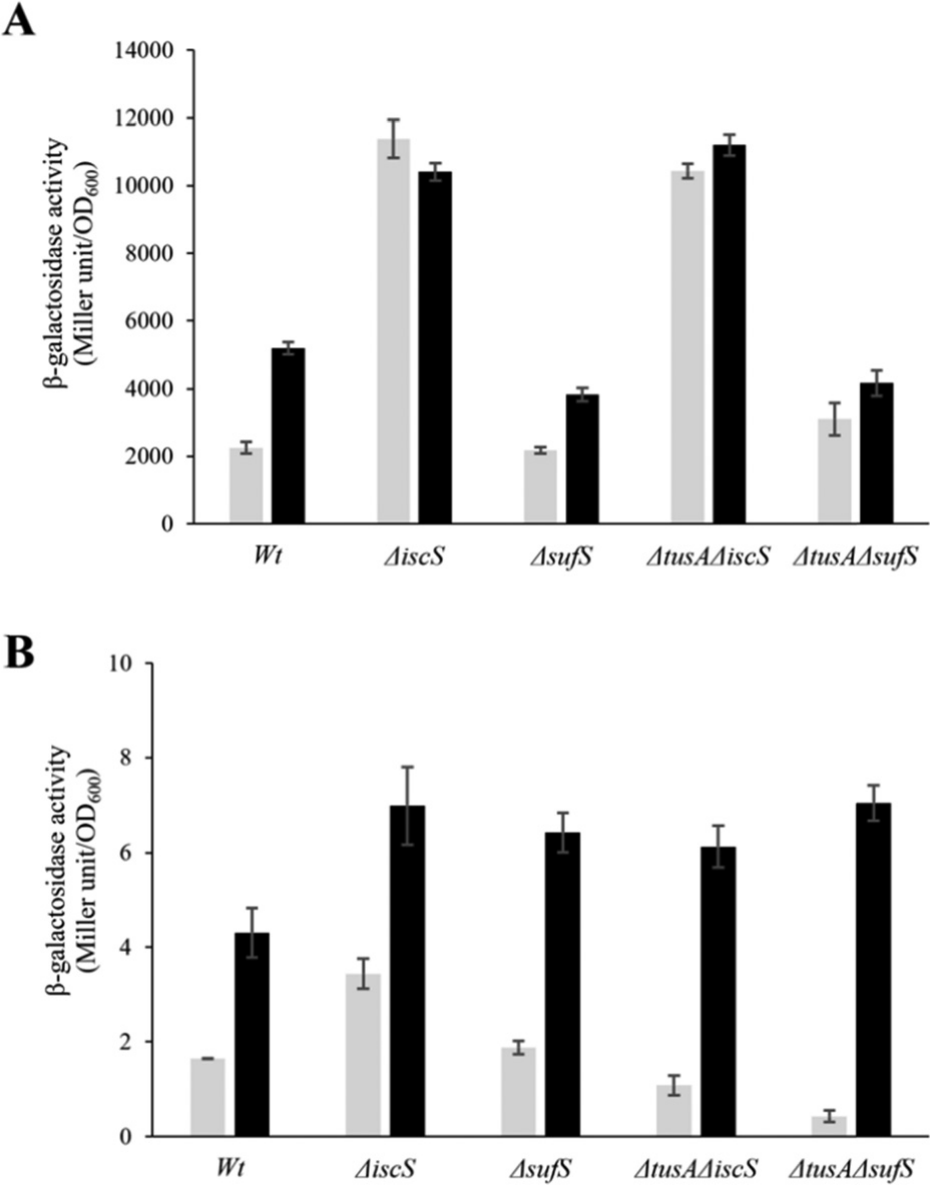
Analysis of the expression of *iscR-lacZ* and *sufA-lacZ* fusions in different *E. coli* strains under aerobic conditions. The expression of *iscR-lacZ* (A) and *sufA-lacZ* (B) fusions was determined as β-galactosidase activity in the *E. coli* BW25113 parental strain (wt), Δ*iscS*, Δ*sufS*, Δ*tusA*Δ*iscS* and Δ*tusA*Δ*sufS* strains. Cells were grown aerobically in LB medium with (black bars) or without (light grey bars) the addition of 100 μM dipyridyl at 37 °C for 7 h. The activity is calculated in Miller Units and related to OD600 nm from 3 independent measurements.

**Fig. 7:**
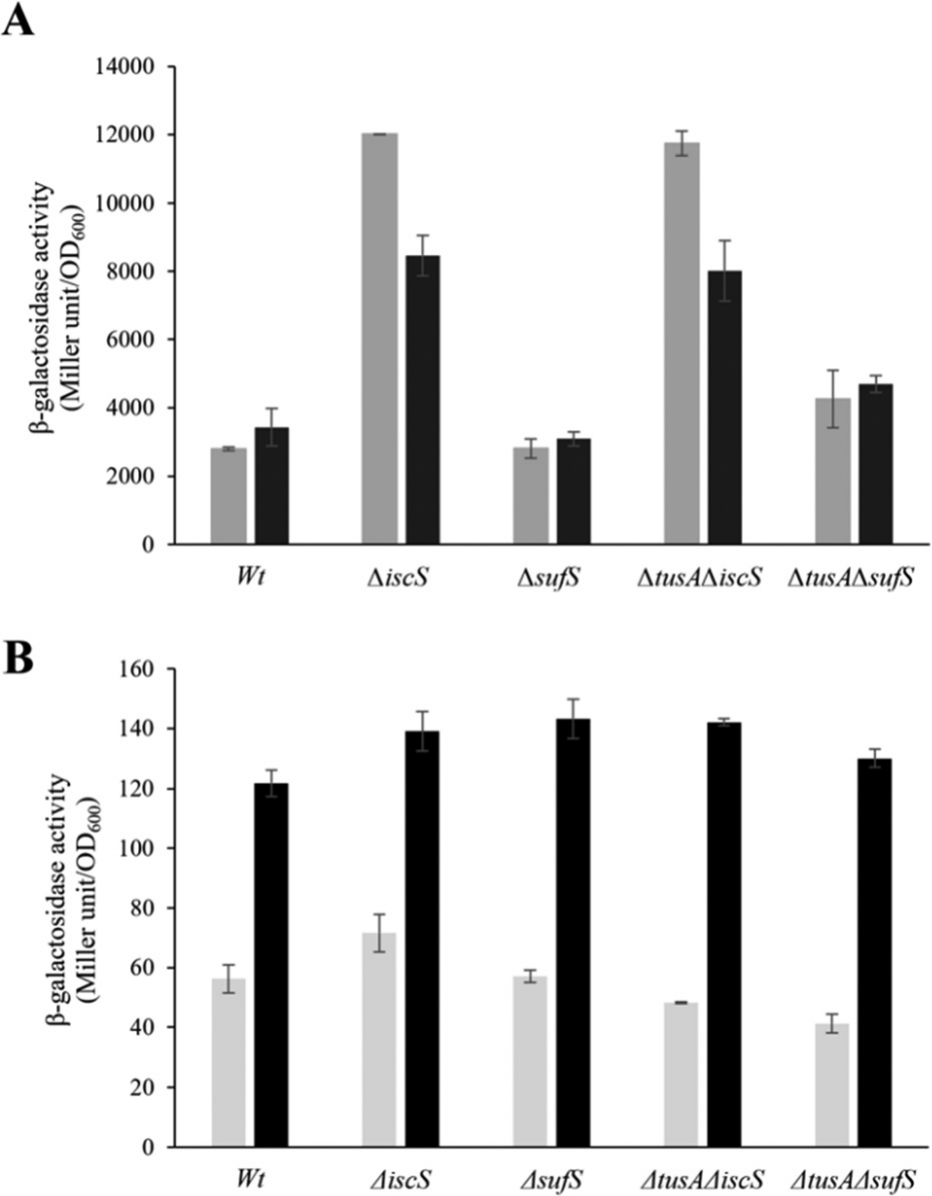
Analysis of the expression of *iscR-lacZ* and *sufA-lacZ* fusions in different *E. coli* strains under anaerobic conditions. The expression of *iscR*-l*acZ* (A) and *sufA-lacZ* (B) fusions was determined as β-galactosidase activity in the *E. coli* BW25113 parental strain (wt), Δ*iscS*, Δ*sufS*, Δ*tusA*Δ*iscS* and Δ*tusA*Δ*sufS* strains. Cells were grown anaerobically in LB medium supplemented with 15 mM potassium nitrate with (black bars) or without (light grey bars) the addition of 100 μM dipyridyl at 37 °C for 7 h. The activity is calculated in Miller Units and related to OD600 nm from 3 independent measurements.

To ensure that the operons are actually expressed under the conditions under which l-cysteine desulfurase activities were measured, β-galactosidase assays were performed for *iscR-lacZ* and *sufA-lac*Z fusions under aerobic (Fig. 6) and anaerobic conditions (Fig. 7), in the presence and absence of dipyridyl as iron chelator (black bars in Fig. 6 and 7). As expected, the *sufA-lacZ* fusion was mainly expressed under aerobic conditions (oxidative stress) and under iron-limiting conditions (presence of dipyridyl).^18^

Under aerobic and iron-limiting conditions, the SUF system is the main producer of Fe–S clusters.^50,51^ Thus, in the Δ*iscS* and Δ*tusA*Δ*iscS* mutant strains, where only the SUF-system is present, higher β-galactosidase activities than for the parental strain were observed. In these *iscS* mutants, activities were lower under low iron (presence of dipyridyl) because of up-regulation of the SUF system leading to the production of more Fe–S clusters and greater cluster incorporation into IscR, down-regulating the ISC-system.

There was essentially no difference between the wild-type and *sufS* mutant strains, consistent with the dominance of the ISC system for the delivery of cluster to IscR.^4,50^ There was also little difference in the *tusA*/*sufS* double mutant, indicating that TusA does not significantly affect cluster delivery to IscR.

Under anaerobic conditions, the ISC-System is the main Fe–S cluster producing system, as obvious from the 100-fold higher expression as compared to the *sufA-lacZ* fusion in the parental strain. Here, *iscR* expression was similar to that under aerobic conditions for wild type under normal iron conditions. Expression increased significantly under low iron, consistent with lower levels of cluster. In the absence of IscS, expression was increased significantly (∼3-fold) because of low cluster loading into IscR, and this was not further affected by low iron, presumably because cluster incorporation in the absence of IscS was not efficient whether iron was present or not. In the *sufS* mutant, levels of *iscR* expression were similar to those of wild-type, consistent with SUF not playing a major role under anaerobic conditions. As observed under aerobic conditions, the *tusA*/*iscS* and *tusA/sufS* double mutants behaved similarly to the corresponding single *iscS* and *sufS* mutants. For *sufA*, there was very low expression under anaerobic conditions, as expected. Expression was higher under low iron, consistent with Fur regulation, but remained low, presumably due to OxyR and low apo IscR regulatory effects.^4,18^

From the above data, it is clear that expression from the *iscR-lacZ*-fusion responds to and reports on the cellular Fe–S cluster content, and is thus suitable as a probe of the effects of cellular conditions on Fe–S cluster assembly activity, as previously reported.^4,25,51^

### The effect of CyaY on the expression of the *iscR-lacZ* fusion

**Fig. 8:**
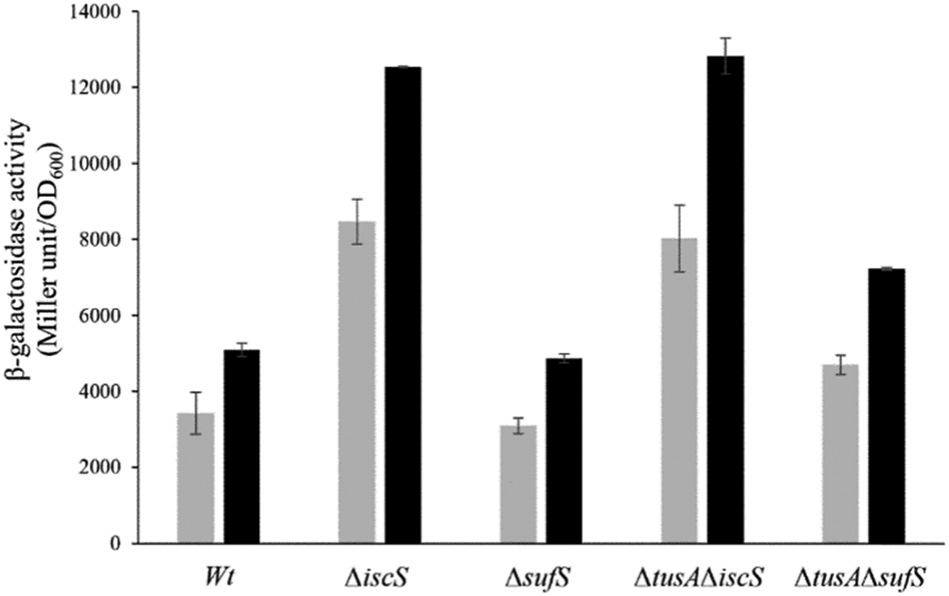
Analysis of the expression of *iscR-lacZ* fusion after overexpression of CyaY in different *E. coli* strains under aerobic conditions in the presence of dipyridyl. The expression of *iscR*-l*acZ* fusion was determined as β-galactosidase activity in the *E. coli* BW25113 parental strain(wt), Δ*iscS*, Δ*sufS*, Δ*tusA*Δ*iscS* and Δ*tusA*Δ*sufS* strains in dependency on CyaY overexpression. Cells were grown aerobically in LB medium with (black bars) or without CyaY (light grey bars) the addition of 20 μM of IPTG, to induce CyaY overexpression, at 37 °C for 7 h. The activity is calculated in Miller Units and related to OD600 nm from 3 independent measurements.

The *iscR-lacZ* fusion was used as a readout for the effect of CyaY on cellular Fe–S cluster synthesis under aerobic conditions where the SUF system plays a key role. Under normal iron conditions, the effect of CyaY on *iscR* expression in the wild-type and mutant strains was not large, with only marginally more expression for each strain when *cyaY* was overexpressed (Fig. S5, ESI†), indicating that IscR had lower cluster levels in the presence of excess CyaY. This included strains lacking SufS, suggesting that excess CyaY has an inhibitory effect on IscS. Under aerobic and low iron conditions, where SUF is upregulated, CyaY had a more significant effect on *iscR* expression, particularly in the Δ*iscS* strains, Fig. 8. Here, SufS is the principal source of sulfide for Fe–S assembly and the presence of excess CyaY inhibited cluster insertion into IscR. This is consistent with the desulfurase activity data (Fig. 5), though the effect was less severe. This suggests that cluster insertion still occurred to some extent, even when overall cellular desulfurase activity was severely decreased. This could either be because the sulfide that is generated by an inhibited SufS is directed specifically to Fe–S assembly, or that some cluster assembly can occur even in the absence of functional IscS/SufS.

## Discussion

It has been reported previously that CyaY influences the activity of IscS, but apparently conflicting conclusions about whether CyaY inhibits or activates the activity of IscS have resulted from *in vitro* and *in vivo* studies. *In vitro* studies have demonstrated an inhibitory effect, while *in vivo* studies indicated a positive effect on ISC Fe–S assembly.^15,17,25^

Adinolfi *et al.* reported that IscX acts as a silencer of CyaY's inhibitory effect *in vitro*.^17^ Both proteins were shown to compete for the same binding site on IscS; interaction with IscX is favored under low-iron conditions, with the result that Fe–S cluster formation proceeds normally. Under higher iron concentrations, CyaY out-competes IscX for binding to IscS and thus exerts its inhibitory effect on Fe–S cluster formation.

However, an *in vivo* study by Roche *et al.*, indicated that both CyaY and IscX contribute positively to ISC Fe–S assembly, and that the effect of CyaY was observed under iron-rich conditions *in vivo*.^25^ However, that study involved a Δ*cyaY* mutant strain whereas here *cyaY* was overexpressed and so different observations are perhaps not unexpected. Indeed, here, an inhibitory effect on IscS activity was observed, consistent with previous *in vitro* studies. Such an effect could be indirect, for example *via* an as yet unidentified regulatory protein. It was proposed that the IscX and CyaY proteins exert their influence by directing sulfur flow towards ISC-mediated Fe–S cluster biosynthesis under “normal” iron conditions, and that apparently contradictory data from *in vitro* and *in vivo* experiments simply reflect the complexity of the *in vivo* situation, and a general lack of broader understanding of the networks of interactions within the cell that affect desulfurase activity.^25^ Our results provide new insight into the role of CyaY and, in addition, bring the TusA protein under consideration as an additional regulatory factor.

IscS can interact with IscU and TusA, at different but overlapping sites, and this competition is the basis for the regulation of sulfur transfer into the different sulfur-requiring pathways.^26^ Here, titration of the IscS–CyaY complex with TusA showed that TusA can displace CyaY from the complex, indicating overlapping binding sites of TusA and CyaY on IscS, with a higher preference for TusA over CyaY. Furthermore, IscS_2_–TusA–CyaY hetero-complexes were identified, consistent with the two IscS molecules of the dimer acting independently in terms of interactions with accessory proteins, as also observed for IscS interacting with IscU and TusA.^26^

The displacement of CyaY from its complex with IscS by TusA would be expected to relieve CyaY inhibition of IscS activity and direct the sulfur flow mainly to tRNA thiolation *in vivo*, as also suggested by Roche *et al.* who investigated the increased sensitivity to lambda phage infection in Δ*cyaY* mutants.^25^ We further considered whether, following its release from IscS, CyaY might be able to interact with the SUF pathway. Firstly, we further investigated the interaction of SufE with SufS, which is well known and structures of the complex are available.^38,40,52^ Taken together, the data presented here show that each SufS dimer contains a pair of high affinity (*K*_d_ ∼ 8 μM) and low affinity (*K*_d_ ∼ 71 μM) binding sites for SufE, reminiscent of the interaction of IscS with IscX.^53^

We note that the *K*_d_ for the higher affinity site measured here is significantly lower than that previously reported value of *K*_d_ ∼ 0.36 μM, determined using surface plasmon resonance measurements.^53^ The reason for this difference is not clear, but it could be associated with the different buffer conditions (250 mM ammonium acetate pH 8 here *versus* 10 mM HEPES pH 7.4, 150 mM NaCl, 3 mM EDTA, 0.005% v/v Surfactant P20 in the earlier study). Binding that is dependent on electrostatic interactions is likely to be sensitive to buffer conditions. We also note that *Bacillus subtilis* SufU–SufS is interchangeable with *E. coli* SufE–SufS, and that *B. subtilis* SufE–SufU complexes have a comparable stability (*K*_d_ = ∼3 μM) to that reported here for the initial complexes formed by the *E. coli* SufS and SufE proteins. Structural data for the *B. subtilis* SufU–SufS complex is also available (PDB: 5XT6).^38^ Based on similarities between SufS and IscS, we propose that the high affinity SufE binding site corresponds to the position of SufU in the available structures. The lower affinity site could possibly be analogous to the accessory protein binding site of IscS (Fig. 2(D)).

It was previously shown that *B. subtilis* frataxin (Fra) is capable of binding to the *B. subtilis* SufU–SufS complex *via* an adjacent binding site.^39^ Importantly, however, binding did not have a significant effect on the desulfurase activity of *B. subtilis* SufU–SufS, nor on *in vitro* Fe–S assembly.^39^ Thus, a significant regulatory role for CyaY resulting from its interaction with SufS has not been considered previously. Titration data presented here following additions of CyaY to SufS alone, or the SufSE complex, demonstrated binding. Although this appeared to be relatively weak, and certainly weaker than the interaction between CyaY and IscS, SufS was still able to compete with IscS for CyaY, suggesting a possible physiological significance. Upon addition of CyaY to SufS/SufE, the (SufE)_3_(SufS)_2_ complex was lost, and (CyaY)(SufE)_2_(SufS)_2_ and, to a lesser extent, (CyaY)_2_(SufE)_2_(SufS)_2_ complexes were observed. This suggests that CyaY recognises and competes with SufE for the second lower affinity binding site present on SufS. Thus, the native MS observations reported here are also consistent with the observations of Blaenburg *et al.*^39^

Both *in vitro* and *in vivo* studies indicated that binding of CyaY to SufS had a dramatic effect on SufS desulfurase activity, reducing it to very low levels. Under iron-replete conditions, this would provide a dual regulation of the SUF system, at the level of expression by Fur and at the protein level by CyaY.^18,50^ Under iron-rich conditions, CyaY interacts with IscS and inhibits its activity *in vivo*.^54^ However, this activity is additionally regulated by the concentration of IscX,^17^ and by TusA, as reported here. Therefore, by considering TusA as an additional regulator of the activity of IscS, the data presented here bridges observations made by Roche *et al.* and Adinolfi *et al.* Furthermore, in the study reported by Roche *et al.*, a role for CyaY in the SUF pathway was excluded. However, by investigating the role of CyaY under iron-limiting conditions, we have identified a clear effect of CyaY on SufS. Indeed, it is under such conditions that SufS is expressed and CyaY is available because it is less likely to be in complex with IscS and IscU. Increases in the concentration of TusA will also lead to displacement of CyaY from IscS, and under low iron, IscX displaces CyaY from IscS,^17^ in both cases increasing its capacity to interact with SufS.

**Fig. 9:**
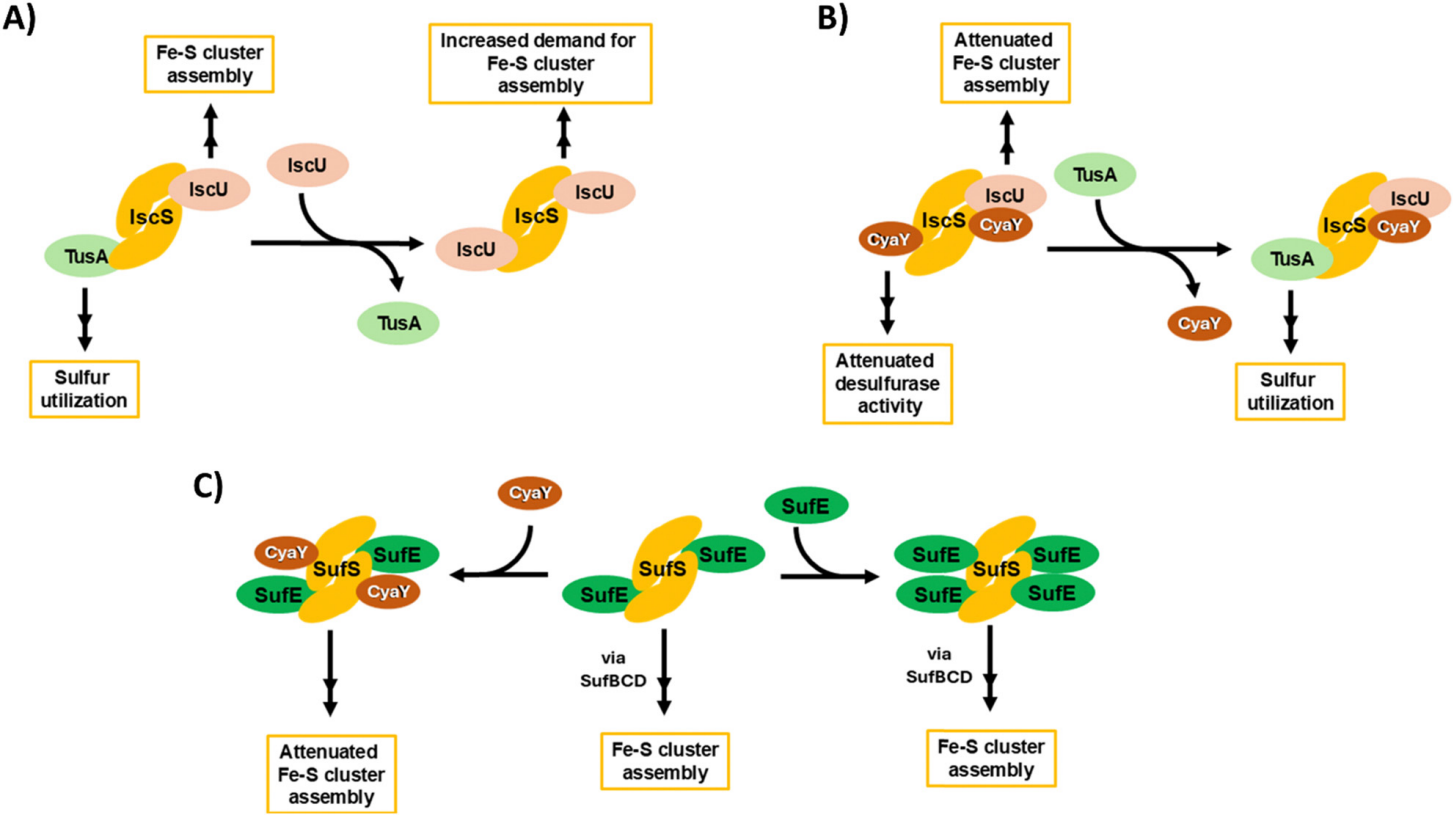
Schematic overview of ISC-mediated sulfur delivery pathways and the interplay between TusA, CyaY and the ISC and SUF Fe–S cluster assembly machineries. (A) IscS contains two independent binding sites for IscU and TusA, allowing for the bifurcation of sulfur utilisation for Fe–S assembly (*via* IscU) and tRNA modification (*via* TusA). Preferential binding of IscU over TusA to IscS limits bifurication, enhancing Fe–S assembly when demand for Fe–S clusters is high. (B) Under iron-replete conditions, CyaY attenuates the desulfurase activity of IscS, reducing the rate of Fe–S assembly. The bifurcation of sulfur utilisation is maintained by TusA, through the displacement of CyaY. (C) Under conditions of iron starvation/oxidative stress, the SUF system supplies Fe–S clusters. CyaY binds to and attenuates the desulfurase activity of SufSE, *via* a secondary binding site. Increased SufE levels may prevent CyaY-mediated attenuation, by competing for the same binding site.

Fig. 9 provides an overview of the complex interplay between TusA, CyaY and the ISC and SUF machineries, connecting the data presented here with the wider field. The complexity begins with IscS, which can direct sulfur along different biosynthetic pathways, including Fe–S cluster assembly, tRNA thiolation and Moco biosynthesis. This involves competition between sulfur-accepting proteins (IscU and TusA are illustrated, Fig. 9(A)). CyaY, the bacterial frataxin homologue, can also bind to IscS, acting as an allosteric modulator (down-regulator) of IscS desulfurase activity and, thus, Fe–S cluster assembly, particularly under high iron conditions. TusA can displace CyaY from its complex with IscS, directing sulfur into tRNA thiolation and Moco biosynthesis (Fig. 9(B)).

CyaY can also bind to SufS, the SUF system cysteine desulfurase, which functions in complex with SufE. SufS can bind up to four SufE molecules per SufS dimer, at high and low affinity sites on each SufS monomer. CyaY appears to bind at the lower affinity site on SufS. As for IscS, CyaY also acts as a negative modulator of desulfurase activity, down-regulating Fe–S cluster assembly (Fig. 9(C)). It is likely that SufE and CyaY compete for the lower affinity site, and so desulfurase activity will depend on relative SufE/CyaY levels. The scheme illustrates that both TusA, in displacing CyaY from IscS, and CyaY itself, are both regulators, either directly or indirectly, of cysteine desulfurase activity.

In summary, the data reported here provide further insight into the protein–protein interactions that play important roles in the ISC and SUF Fe–S cluster assembly machineries of *E. coli*. Most notably, an interaction between CyaY and SufS was demonstrated, which *in vivo* resulted in the deactivation of SufS desulfurase activity. The effect on Fe–S cluster assembly was also found to be significant, though less severe. Thus, we conclude that CyaY is an allosteric regulator of both IscS and SufS desulfurases, and hence both the ISC and SUF Fe–S cluster biogenesis systems of *E. coli*.

## Experimental procedures

### Bacterial strains, media, and growth conditions

BW25113 (referred to as wild-type strain) and the isogenic mutant strains Δ*iscS* and Δ*sufS* (Keio collection) were obtained from the National BioResource Project (National Institute of Genomics, Japan).^55^ The double mutants Δ*tusA*Δ*iscS* and Δ*tusA*Δsuf*S* were constructed by deleting the already present KANA-cassette using the plasmid pCP20 and reintroduced the KANA-cassette in the second gene of interest using the plasmid pKD46. Amplified DNA-fragments were used by the recombinase (expressed by pKD46). Each strain was verified by colony verification PCR.

For protein expression using T7 promoter plasmids, DE3 (lacZ promoter + T7 polymerase gene) was inserted using the λDE3 Lysogenization Kit (Novagen – Sigma-Aldrich) in the wild-type strain and mutants to overexpress the protein under study. *E. coli* cultures were grown in LB medium under aerobic conditions at 37 °C for 7 h and, when required, kanamycin (50 μg ml^−1^) and ampicillin (150 μg ml^−1^) were added to the medium.

### Purification of IscS, SufS, CyaY, SufS and TusA

*E. coli* BL21 DE3 competent cells were transformed with the respective overexpressing plasmid: IscS (pSL209, Amp^R^), CyaY-(His)_6_ (pET15b, Amp^R^ or pMB10, Amp^R^), SufS-(His)_6_ (pSL213, Amp^R^), SufE-(His)_6_ (pET15b, Amp^R^) or TusA (pJD34, Amp^R^). Expression and purification of the proteins were carried out following previously published procedures.^26^ The purified SufS and CyaY proteins were further subjected to (His)_6_-tag cleavage by overnight incubation of the purified proteins with 5 mg ml^−1^ of thrombin at 4 °C and further passage down a Ni^2+^–agarose column to remove the (His)_6_-tag. Protein concentrations were quantified using the Bradford Reagent Coomassie Plus Protein Assay Reagent (Thermo) and bovine serum albumin (BSA) as a standard, following the manufacturer's instructions.

### Quantification of l-cysteine desulfurase activity

The activity of SufS in the presence of various combinations of SufE and CyaY was quantified after the release of sulfide as methylene blue following published procedures.^56^ Here, 30 μM of SufS or 30 μM of SufS plus 90 μM of SufE were incubated, with or without 90 μM of CyaY and in the absence or presence of 50 μM of FeCl_3_, for 10 min at 30 °C in the presence of 1 mM DTT and 1 mM l-cysteine. The reaction was stopped and each product quantified by using a standard sulfide calibration curve (0–200 μM sulfide). One unit of activity is defined as the amount of enzyme producing 1 μmol of sulfide/min.

### Quantification of the total H_2_S production from cell extracts

Strains were grown aerobically for 7 h in LB medium supplemented with or without 20 μM of IPTG, and with or without 100 μM of 2,2′-dipyridyl, as needed. The cells were harvested and washed with tris/HCl 50 mM pH 7.5. Total l-cysteine desulfurase activities of crude cell extracts obtained by sonification were determined using the methylene blue assay following published procedures (6). The supernatant of cell extracts after centrifugation were accurately diluted and incubated with 1 mM DTT and 1 mM l-cysteine for 10 min at 30 °C. The reaction was stopped and sulfide levels quantified by using a standard sulfide calibration curve (0–200 μM sulfide). One unit is defined as the amount of enzyme producing 1 μmol of sulfide/min.

### Mass spectrometry under non-denaturing conditions

Proteins (IscS, TusA, CyaY, SufS, SufE) were exchanged into 250 mM ammonium acetate, pH 8.0 using PD mini-Trap G25 columns (Cytiva) and the concentration determined using the appropriate calculated extinction coefficients. Samples (200 μL) were prepared immediately prior to use, by dilution, and contained ∼8 μM IscS or SufS (∼4 μM dimer) together with appropriate ratios of other proteins (TusA, CyaY, SufE). Samples were infused directly, *via* syringe pump, into the source of a Waters Synapt XS (Waters Corp.) or a Bruker micrOTOF-QIII (Bruker Daltonics) mass spectrometer, operating in the positive mode with a capillary voltage of 3,500 V. Optimization of the experimental conditions for the transmission of dimeric IscS and associated complexes was achieved by increasing the cone-voltage to 150 V (135 V isCID on the Bruker instrument). Other parameters were optimised according to Laganowsky *et al.*,^57^ behaving in broadly similar ways on Waters and Bruker instruments^58^ MS instruments were calibrated with sodium iodide (Waters Corp.) and/or ESI-L low concentration tuning mix (Agilent Tech.). Data were acquired over the *m*/*z* range 3000–8000 for 5 min and then averaged.

Processing and analysis of MS experimental data were carried out using Waters Mass Lynx v4.2 (Waters Corp.) or Bruker Compass Data analysis v4.1 (Bruker Daltonik). Neutral mass spectra over the 90–140 kDa range were generated using the maximum entropy deconvolution algorithm of each analysis suite, or UniDec.^59^ The high *m*/*z* range in which these large multi protein complexes (*e.g.* (SufS)_2_(SufE)_2_) and their adducts were detected negatively affected mass resolution for both the Waters and Bruker spectrometers, and, in general, low mass adducts (*e.g.*^32^S, ^56^Fe, *etc.*) could not be unambiguously identified. Nevertheless, the protein constituents of complexes could be unambiguously determined. Fractional intensities were calculated for each species present during titration from total ion counts, and data were fitted using the program Dynafit (Biokin), as previously described.^60^ Routine LC–MS was used to check the mass of apo proteins under denaturing conditions, as previously described.^34^

## Author contributions

PO, JCC, NLB and SL conceived the study and contributed to experimental design. AL and PO contributed to protein production and purification. PO and JCC analysed the data. PO, JCC, NLB and SL wrote the paper. All authors approved the final version of the manuscript.

## Conflicts of interest

There are no conflicts to declare.

## Supplementary Material

CB-005-D4CB00225C-s001

## Data Availability

Date supporting the conclusions of this study are available in the main text with additional experimental data given in the ESI.†
