# Supplementary material for: CyaY and TusA regulate ISC- and SUF-mediated l-cysteine desulfurase activity
Source: RSC Chem Biol. 2024 Sep 27;5(11):1165–76. doi: 10.1039/d4cb00225c (PMC11446229; doi:10.1039/d4cb00225c)
Supplement: CB-005-D4CB00225C-s001 [file CB-005-D4CB00225C-s001.pdf]

Supplementary Information (SI) for RSC Chemical Biology.
This journal is © The Royal Society of Chemistry 2024

SUPPORTING INFORMATION

CyaY and TusA regulate ISC- and SUF-mediated L-cysteine desulfurase activity

*Paolo Olivieri[1], Jason C. Crack[2] Angelika Lehmann[1], Nick E. Le Brun[2], and Silke Leimkühler[1]**

Supporting Figures

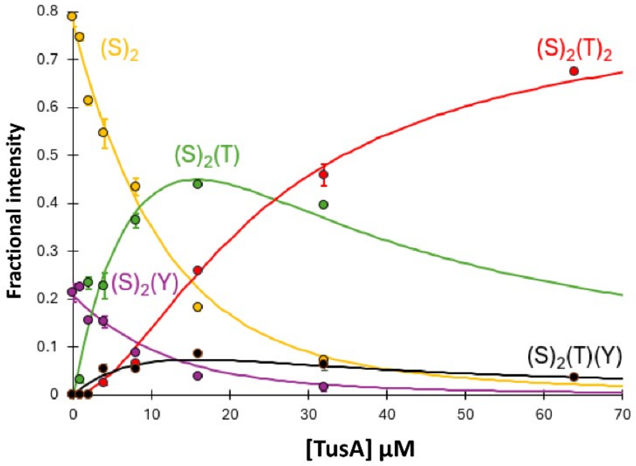

Figure S1. ESI-MS investigation of complex formation between CyaY-IscS and TusA. Plots of relative intensity of various complexes, as indicated, as a function of TusA concentration. Solid lines show fits of the data to a simple competitive binding model for 1-2 CyaY per IscS dimer.

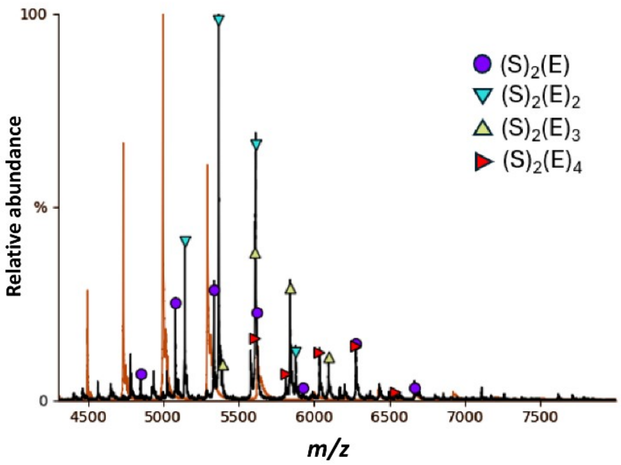

Figure S2. ESI-MS investigation of complex formation between SufS and SufE. *m/z* spectrum of SufS before (brown spectrum) and after (black spectrum) the addition of SufE at a 4:1 ratio. Charge states associated with multiple SufE-SufS complexes are indicated. The deconvoluted spectrum is shown in Figure 2A.

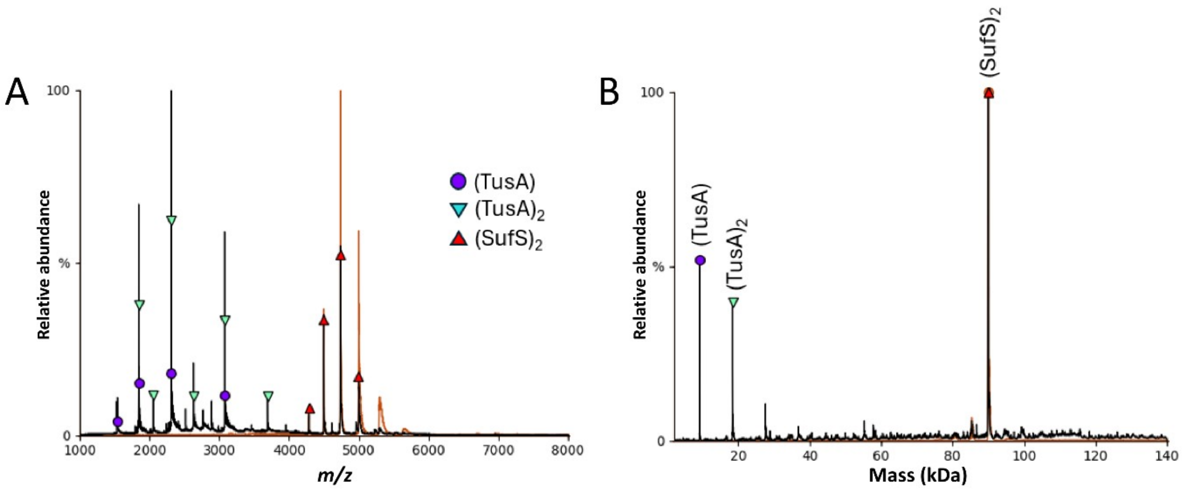

Figure S3. TusA does not bind SufS. A*)* *m/z* spectrum of SufS in the presence (black spectrum) and absence (red spectrum) of TusA at a 2:1 ratio. Charge states form SufS-TusA complexes are absent from the spectrum. The spectrum is dominated by monomeric TusA (purple circles) and dimeric SufS (red triangles). **B)** Deconvoluted spectrum showing dimeric SufS (red triangle) and monomeric (purple circle) and dimeric (blue triangle) TusA, but not SufS-TusA complexes, consistent with previous observations [1].

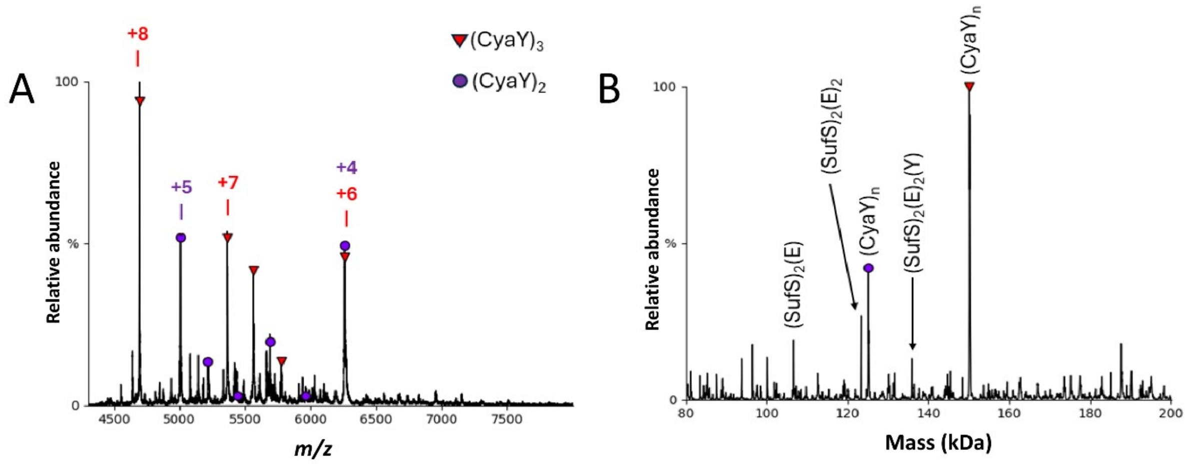

Figure S4. ESI-MS investigation of complex formation between SufE-SufS and CyaY. A) *m/z* spectrum of SufS-SufE-CyaY complexes at an 8:1 ratio of CyaY to SufS-SufE, showing CyaY-mediated ion suppression. The spectrum is dominated by dimeric (purple circle) and trimeric (red triangles) CyaY charge states, which overlap with complexes of interest (charge numbers for CyaY multimers are indicated). Other marked charge states, without charge numbers, are misassigned to CyaY multimers during deconvolution [2]. **B)** Deconvoluted spectrum showing harmonic effects from CyaY multimers. The 4th harmonic of trimeric CyaY (red triangle) and the fifth harmonic of dimeric CyaY (blue circle) dominate the deconvoluted spectrum suppressing signals from other complexes.

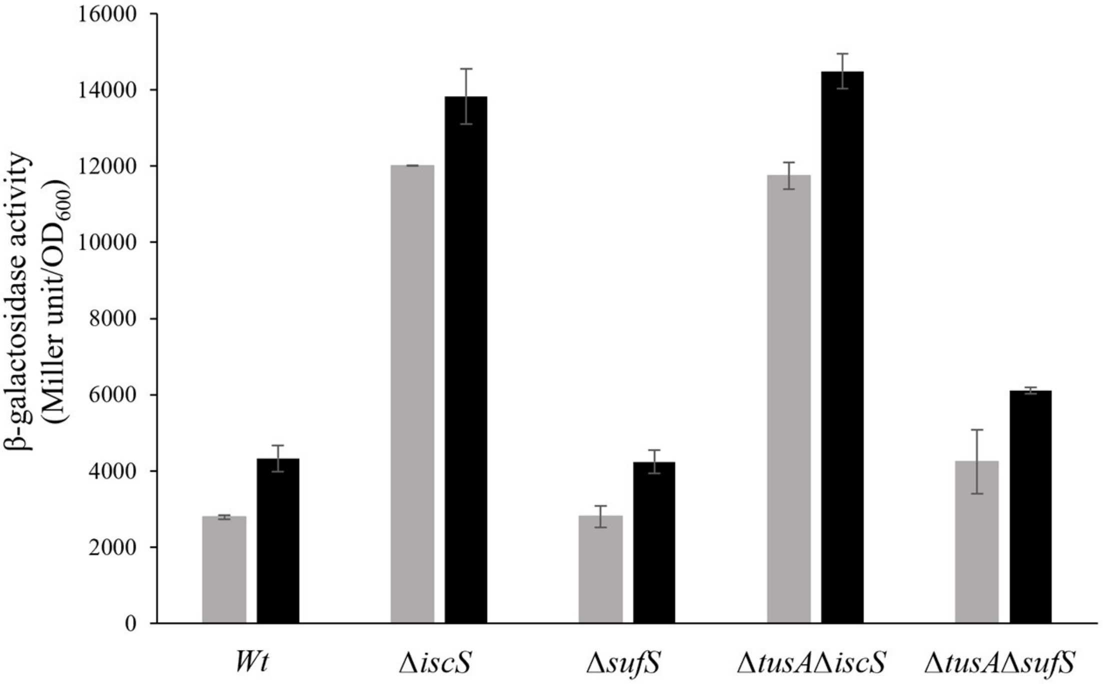

Figure S5: Analysis of the expression of *iscR-lacZ* fusion after overexpression of CyaY in different *E. coli* strains under aerobic conditions in the absence of dipyridyl. The expression of *iscR*-l*acZ* fusion was determined as β-galactosidase activity in the *E. coli* BW25113 parental strain (Wt), *ΔiscS, ΔsufS*, *ΔtusAΔiscS* and *ΔtusAΔsufS* strains in dependency on CyaY overexpression. Cells were grown aerobically in LB medium with (black bars) or without CyaY (light grey bars) the addition of 20 μM of IPTG, to induce CyaY overexpression, at 37 °C for 7 h. The activity is calculated in Miller Units and related to OD600 nm from 3 independent measurements.

References

1. M. Bühning, A. Valleriani and S. Leimkühler, The Role of SufS Is Restricted to Fe-S Cluster Biosynthesis in *Escherichia coli*, *Biochemistry*, 2017, **56**, 1987-2000.
2. M. Bern, T. Caval, Y. J. Kil, W. Tang, C. Becker, E. Carlson, D. Kletter, K. I. Sen, N. Galy, D. Hagemans, V. Franc and A. J. R. Heck, Parsimonious Charge Deconvolution for Native Mass Spectrometry, *J Proteome Res*, 2018, **17**, 1216-1226.
